# Tubulointerstitial nephritis and uveitis (TINU) syndrome: a case series

**DOI:** 10.1186/s12348-025-00500-x

**Published:** 2025-07-18

**Authors:** Richard Farnan, John Stokes, Rob Casey, Aisling McGlacken Byrne, Sean Leavey

**Affiliations:** 1 University Hospital Galway, Galway, Ireland; 2 University Hospital Waterford, Waterford, Ireland

**Keywords:** Tubulointerstitial nephritis, Uveitis, TINU syndrome, Case series

## Abstract

**Purpose:**

Tubulointerstitial nephritis and uveitis (TINU) syndrome, characterised by the co-occurrence of tubulointerstitial nephritis and uveitis in the absence of other systemic diseases, presents a diagnostic challenge due to its non-specific symptoms. This case series aims to shed light on TINU syndrome’s clinical features, underlying causes, and management strategies. The prevalence of TINU syndrome is relatively low with estimates of 3.5 cases/ million people with an incidence of 0.2 cases/ million/ year and it often goes underdiagnosed. The ratio of males to females reported was 4:1. Histological confirmation through renal biopsy is crucial, while systemic conditions such as systemic lupus erythematosus, tuberculosis, sarcoidosis, and Sjogren’s disease should be excluded. In most cases, TINU appears to be an idiopathic immune-mediated process, but it may be precipitated by drugs or infections. Antibiotics and non-steroidal anti-inflammatory drugs (NSAIDs) have been associated with some cases of TINU syndrome. Genetic markers, particularly HLA subtypes, have shown a strong association with TINU syndrome. Uveitis which is characterised by intraocular inflammation is a common feature of TINU syndrome. Anterior uveitis, the most prevalent form, presents with symptoms such as red eye, eye pain, photophobia, and keratic precipitates which are subdivided into granulomatous and non-granulomatous. Corticosteroids represent the primary treatment for ocular inflammation, while immunosuppressant medications may be employed for treatment of nephritis.

**Observations:**

Through the analysis of five TINU syndrome cases, this case series provides insights into the clinical presentations, laboratory findings, and biopsy results of patients with TINU syndrome. The cases include individuals with associated systemic conditions such as asthma, psoriasis and hyperthyroidism.

**Conclusion and importance:**

In conclusion, TINU syndrome is a rare condition characterised by the simultaneous occurrence of tubulointerstitial nephritis and uveitis. Increased awareness among healthcare professionals is necessary for early recognition and appropriate management of this syndrome. Further research is needed to elucidate the pathogenesis, optimise diagnostic criteria, and explore more targeted therapeutic approaches for TINU syndrome.

## Learning points

• The association between uveitis and certain systemic diseases exists and may manifest the non-constitutional symptoms before the onset of ocular disease.
• Certain drug classes and infections have been implicated in TINU syndrome and should be considered at the time of diagnosis.
• Renal biopsy provides the definitive diagnosis for tubulointerstitial nephritis.
• TINU is likely an underdiagnosed condition and the necessity for nephrologists to include an ocular dimension to their history taking is therefore important.
• Ophthalmology doctors should include a nephrology work-up in cases of sudden bilateral uveitis as part of their systemic work-up.

## Introduction

Tubulointerstitial nephritis (TIN) and uveitis was initially described by Dobrin et al. in 1975 [1]. The condition holds no specific diagnostic criteria but is defined as the occurrence of tubulointerstitial nephritis and uveitis in a patient in the absence of other systemic diseases that can cause interstitial nephritis or uveitis [2]. The exact cause of TINU is unknown but it is thought to be an autoimmune mediated event that may be related to infection, certain medications or idiopathic in nature [2].

Research has shown that the prevalence of TINU in uveitis centres is low but the syndrome is also under-diagnosed [3]. TIN in isolation is a potentially life-threatening condition and is diagnosed histologically as interstitial oedema with inflammatory cell infiltrates [4]. Patients with unexplained acute kidney injury (AKI) events or reduced estimated glomerular filtration rate (eGFR) should consider TIN as a noteworthy differential. The precise array of symptoms associated with TIN are largely non-specific. A renal biopsy is therefore required to confirm diagnosis. In addition to this, noteworthy systemic conditions must be excluded including systemic lupus erythematous (SLE), Tuberculosis (TB), sarcoidosis and Sjogren’s disease.

**Fig. 1:**
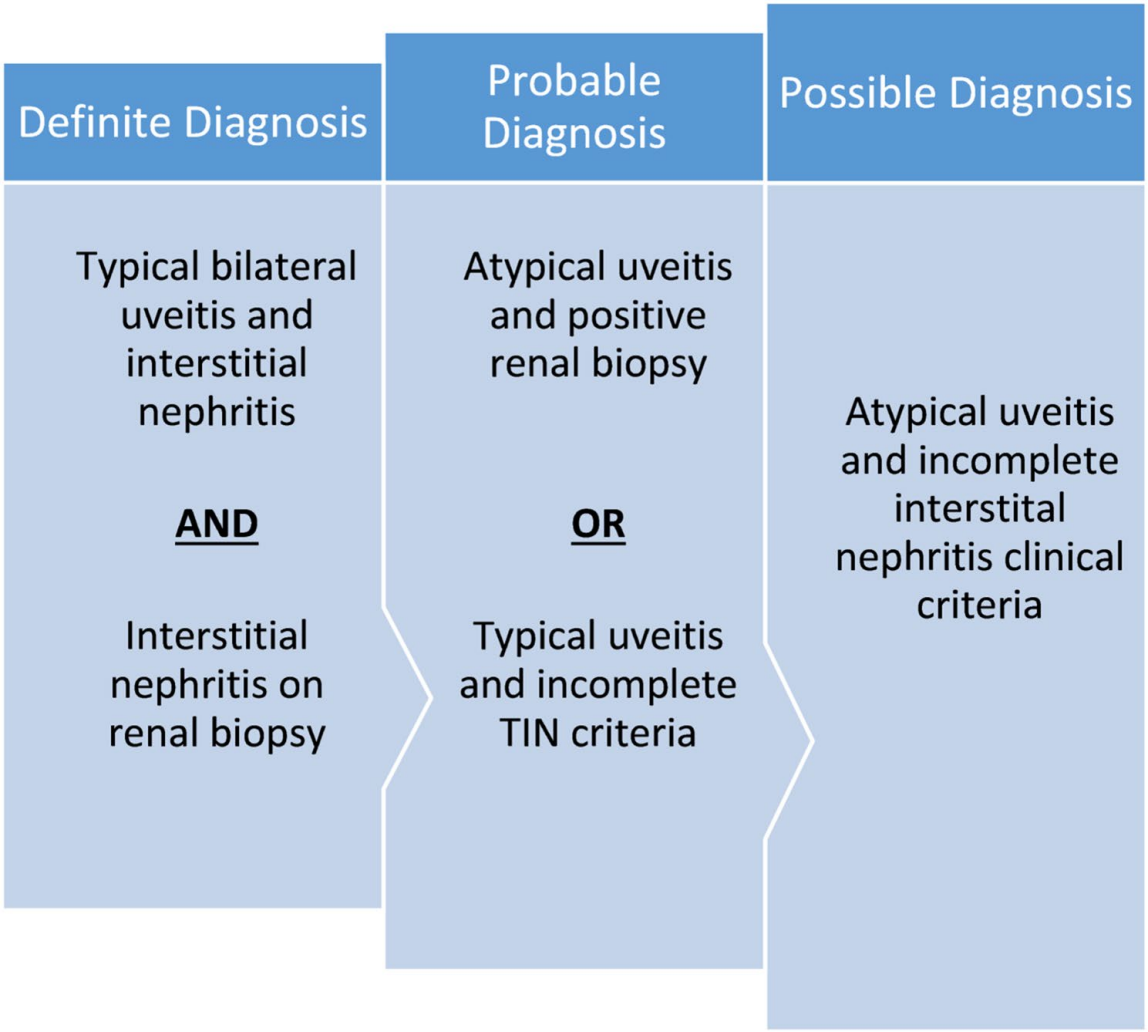
Diagnostic criteria of tubulointerstitial nephritis and uveitis syndrome (TINU syndrome)

Mandeville et al. devised diagnostic criteria for the classification of patients with TINU which is illustrated in the fig. 1 shown below.

Interstitial nephritis clinical criteria

1. Abnormal renal function.
2. Abnormal urinalysis.
3. Systemic illness ≥ 2 weeks including fever, weight loss, lab abnormalities.

The purpose of this case series is to raise awareness of this rare clinical entity and to highlight the importance of screening for uveitis in patients diagnosed with interstitial nephritis in a level three hospital in the south east of Ireland. In this series, five clinical case studies of TINU syndrome are presented and clearly articulate the associated between interstitial nephritis and uveitis.

### Clinical cases

#### Case 1

A 42 year old female presented to eye casualty in University Hospital Waterford (UHW) with acute bilateral red eye of 7 day duration. She gave a history significant for malaise and anorexia of one month duration with no previous episodes reported. Background history significant for asthma. She was untreated in the lead up to initial presentation.

Ophthalmology findings were significant for conjunctival injection bilaterally, inferior non-granulomatous keratic precipitates bilaterally, + 3 cells in the anterior chamber bilaterally (SUN criteria) and normal intraocular pressures. Her presenting visual acuity was 0.3 LogMAR to the right eye and 0.4 LogMAR to the left eye unaided. A diagnosis on non-granulomatous anterior uveitis was made. The SUN Working Group commenced in 2004 when 50 uveitis specialists came together to standardize the approach to classifying uveitis clinical cases [5]. A consensus on the anatomic classification was reached detailing structural components of uveitis, anterior chamber grading, anterior chamber flare and inflammation severity [5].

Laboratory evaluation showed Creatinine level of 333 µmol/L(eGFR-15 ml/min/1.73m^2^), urea 8.4 mmol/L, free thyroxine 19.2 pmol/L, TSH 0.062 mLU/L, Anti Thyroid Microsomal Ab-Negative, Anti Thyroglobulin Ab-Negative. Serum ACE- 33 (8–52) U/L. Urinary Protein/creatinine ratio (U PCR) − 63 mg/mmol (0–15). Anti- Nuclear Antibody (ANA)-negative, Anti DsDNA-negative, Anti-Ro/La-negative, Anti-Neutrophil Cytoplasmic Ab (ANCA) -negative.

A renal biopsy was performed following nephrology input which showed features consistent with active chronic tubule interstitial nephritis.

The presence of uveitis and TIN justified a diagnosis of TINU syndrome. Infective and systemic causes were ruled out and included: hepatitis B and C, Toxoplasma gondii, Epstein-Barr, cytomegalovirus, brucellosis, syphilis, systemic lupus erythematous (SLE), Tuberculosis (TB), sarcoidosis and Sjogren’s disease.

The patient was subsequently managed on oral steroids in the form of prednisolone (1 mg/kg/day) as monotherapy. After 2 months of commencing treatment her creatinine improved to 120 µmol/L (eGFR-50 ml/min/1.73m^2^). She was followed for 13 years in our nephrology clinic and had no recurrence during her follow up and was discharged from our clinic with a creatinine of 92 µmol/L (eGFR-63 ml/min/1.73m^2^). At this point, her visual acuity had improved to 0.2 LogMAR in the right eye and 0.3 LogMAR in the left eye.

#### Case 2

A 57 year old gentleman presented to hospital feeling generally unwell with a red left eye associated with significant pain on eye movement. Three weeks previously he had been admitted to a level 3 hospital with night sweats, right renal angle pain and rigors. He was treated as a urinary tract infection and his antibiotic regimen included Clarithromycin and Cephalexin. His creatinine on discharge from that hospital was 206 µmol/L (eGFR-32 ml/min/1.73m^2^). His baseline creatinine prior to that hospital admission was 100–110 µ mmol/L (eGFR-68-76 ml/min/1.73m^2^). His medical history was significant for psoriasis.

Labaratory evaluation showed a Creatinine level of 181 µmol/L(eGFR-37 ml/min/1.73m^2^), urea 6.4 mmol/L, TSH 1.85 mLU/L. Anti- Thyroid Microsomal Ab-negative, Anti Thyroglobulin Ab.-Positive titre 1:100. ANA-Negative, ANCA-negative. U PCR-36 mg/mmol. Serum ACE-19 U/L (8 to 52). Urine- AFB negative and TB culture negative. The systemic conditions which include SLE, TB, sarcoidosis and Sjogren’s disease were ruled out.

He was seen in eye casualty and ophthalmology findings were reported as fine non-granulomatous keratic precipitates in the left corneal endothelium and + 2 cells in the anterior chamber along with left posterior synechiae. His presenting visual acuity was 0.4 LogMAR bilaterally unaided. Fundal examination was unremarkable bilaterally with no posterior segment involvement noted. A clinical diagnosis of left non-granulomatous anterior uveitis was made.

The electrolyte results prompted nephrology input with subsequent biopsy showing features consistent with severe acute tubulointerstitial nephritis. This patient was managed with oral steroids in the form of prednisolone (1 mg/kg/day). After 12 months of follow up creatinine reached a nadir of 108umol/L (eGFR-69 ml/min/1.73m^2^). His visual acuity had improved to 0.2 LogMAR bilaterally unaided. He was discharged from clinic following 3 years of follow up with a U PCR-15 mg/mmol and creatinine-115 µmol/L (eGFR-64 ml/min/1.73m^2^).

#### Case 3

A 45 year gentleman was referred to the Acute Medical Assessment Unit with a 6 week history of ‘flu-like’ symptoms. He reported generalised myalgia; weight loss of 7 kg; night sweats, fevers and fatigue. He also reported new onset bilateral red eye, photophobia and pain affecting both eyes.

His medical history was significant for type 2 diabetes mellitus and dyslipidaemia. His medications included Metformin, Gliclazide, Sitagliptin and Pravastatin.

Initial investigations performed showed a creatinine level of 231 µmol/L (eGFR-30 ml/min/1.73m^2^), urea 15.6 mmol/L, free thyroxine 44.9 pmol/L, TSH 0.008 mLU/L. ANA-Pos (very weak), Anti Thyroid Microsomal Ab-Negative, Anti Thyroglobulin Ab-Negative, Anti dsDNA Ab- Negative, Anti SSA(Ro) Ab-Negative, Anti SSB(La) Ab-Negative, ANCA-negative and U PCR-51 mg/mmol.

He was subsequently reviewed in eye casualty and ophthalmology findings reported right conjunctival injection, + 3 cells in the right anterior chamber and + 1 cells in the left anterior chamber. Intraocular pressures were 6 mm Hg in the right eye and 10 mm Hg in the left eye. His initial visual acuity was 0.3 LogMAR bilaterally with glasses. Fundal exam was normal and there was no posterior synechiae noted. The posterior segment exam findings were non-contributory. A diagnosis of non-granulomatous bilateral anterior uveitis was made. The systemic conditions which include SLE, TB, sarcoidosis and Sjogren’s disease were ruled out.

A renal biopsy was performed, and it demonstrated features of active moderate acute tubulointerstitial nephritis most consistent with allergic aetiology.

He was commenced on oral prednisolone (1 mg/kg/day). His newly diagnosed hyperthyroidism was managed by our endocrinology colleagues with carbimazole 20 mg once daily orally.

He was followed up in Nephrology clinic for 4 years without any recurrence of disease and was discharged from clinic with a creatinine of 94 µmol/L (eGFR-86 ml/min/1.73m^2^). His visual acuity remained stable at 0.3 LogMAR bilaterally with the aid of glasses.

#### Case 4

A 71 year old gentleman presented to the Emergency Department with a 4 week history of feeling generally unwell with nausea, fatigue, weight loss and had bilateral red eyes with photophobia. His background history was significant for type 2 diabetes mellitus, hypertension and dyslipidaemia. His ophthalmology history was significant for primary open angle glaucoma (POAG).

Laboratory findings reported a Creatinine level of 317 µmol/L (eGFR-17 ml/min/1.73m^2^), urea 8.5 mmol/L, free thyroxine 15.9 pmol/L, TSH 3.6 mLU/L. ANA-pos (very weak). U PCR-14 mg/mmol. HbA1c- 48 mmol/mol. The systemic conditions which include SLE, TB, sarcoidosis and Sjogren’s disease were ruled out.

He was referred to eye casualty and ophthalmology findings were relevant for bilateral conjunctival infection, + 1 cells in the right eye and + 2 cells in the left eye. His presenting visual acuity was recorded as 0.2 LogMAR in the right eye and 0.3 LogMAR in the left eye unaided. The posterior segment exam was non-contributory. These clinical findings were in keeping with a diagnosis of anterior uveitis.

He underwent a renal biopsy which demonstrated tubulointerstitial nephritis with the presence of eosinophils suggestive of allergic aetiology.

This patient was initially managed with oral steroids for 6 months using prednisolone (1 mg/kg/day) and was subsequently commenced on azathioprine (1.5 mg/kg/day). Following 3 months of commencing treatment his creatinine improved to 145 µmol/L (eGFR- 44 ml/min/1.73m^2^). The most recent visual acuity was recorded as 0.2 LogMAR bilaterally unaided. This patient was followed up in nephrology clinic until he passed away 4 years later.

#### Case 5

A 59 year old gentleman was referred to nephrology clinic with a rise in his serum creatinine 160 µmol/L (eGFR-43 ml/min/1.73m^2^) noted 3 months previously by his GP. He had a previous baseline creatinine of 70–90 µmol/L (eGFR-85-102 ml/min/1.73m^2^). He denied any constitutional symptoms and reported he otherwise felt well. He had a history of venous thromboembolism (VTE) with weak positive anti-phospholipid antibody tests and was commenced on warfarin approximately two years previously.

Laboratory results showed a creatinine level of 160 µmol/L (eGFR-43 ml/min/1.73m^2^), urea 7.6 mmol/L, free thyroxine 14.3 pmol/L, TSH 5 mLU/L. HBs Ag-Not Detected, Anti-HCV-Not Detected, HIV Ag/Ab -Not Detected, Urinary Albumin/Creatinine Ratio-25 mg/mmol (0.0 to 2.5), ANA- Pos (very weak), Anti- Proteinase 3 Ab -negative, Anti Myeloperoxidase Ab-negative, Anti GBM Ab-Negative.

He had a renal biopsy performed which demonstrated low grade interstitial nephritis. His anticoagulation was changed to apixaban prior to renal biopsy for ease of management. The systemic conditions which include SLE, TB, sarcoidosis and Sjogren’s disease were ruled out.

At a subsequent clinic appointment, on receipt of renal biopsy results, it was noted that he had bilateral red eyes. The patient had been attending our Nephrology clinic for 6 months at this point. He also reported that this symptom had been ongoing for approximately 2 years and he attributed it to occupational exposure to dust.

He was reviewed in eye casualty and ophthalmology findings were significant for bilateral conjunctival injection with + 0.5 cells in the right anterior chamber and + 2 cells in the left anterior chamber. Intraocular pressures were 23 mm Hg in the right eye and 20 mm Hg in the left eye. His initial visual acuity was 0.4 LogMAR in the right eye and 0.3 LogMAR in the left eye.There was no posterior synechiae noted and fundal exam was non-contributory. The posterior segment exam was unremarkable. A diagnosis of bilateral anterior uveitis secondary to tubulointerstitial nephritis was made. The patient was treated with topical steroids and a cycloplegic agent and showed an improvement in symptom control and anterior chamber activity at the two week follow-up period. The final visual acuity was 0.2 LogMAR bilaterally following steroid treatment.

He has been managed with oral steroid prednisolone (1 mg/kg/day) in the Nephrology clinic. His most recent creatinine was 148mmol/L (eGFR-47 ml/min/1.73 m²) which was 6 weeks after commencing therapy.

## Discussion

This paper presents five cases of TINU syndrome in a major teaching hospital in the south east of Ireland with consideration shown to each presentation, management and follow-up plans.

The ratio of males to females reported was 4:1. Two of the patients in this case series presented with ocular symptoms initially while the other three presented with classical nephrology presentations (Table 1). All five patients received histology input with renal biopsy reports definitely proving interstitial nephritis. From an ophthalmology standpoint, the clinical presentation was similar with four out of five cases reporting bilateral red eye on examination. All of the cases were diagnosed as having non-granulomatous anterior uveitis with no intermediate, posterior, panuveitis in any of the cohort as diagnosed clinically by ophthalmologists.

**Table 1:** Patient characteristics for TINU cohort

	Case 1	Case 2	Case 3	Case 4	Case 5
Gender	Female	Male	Male	Male	Male
Age (years)	42	57	45	71	59
Ocular clinical presentation	Acute bilateral red eye.	Unilateral red eye, blurring of vision.	Bilateral red eye, photophobia, eye pain.	Bilateral red eye.	Bilateral red eye, photophobia.
First renal symptoms	-	-	+	+	+
First ocular symptoms	+	+	-	-	+/-
Histological diagnosis	+	+	+	+	+
Ocular treatment	Topical steroids, mydriatics	Topical steroids, mydriatics	Topical steroids, mydriatics	Topical steroids, mydriatics	Topical steroids, mydriatics
Systemic Treatment	Oral steroid	Oral steroid	Oral steroid	Oral steroid	Oral steroid
Known systemic conditions	Asthma	Psoriasis	Type 2 Diabetes Mellitus, hyperlipidaemia	Type 2 Diabetes Mellitus, hypertension, hyperlipidaemia	VTE (Venous Thromboembolism)
Creatinine at presentation (µmol/l)	333	181	231	317	160
Length of time of follow up with nephrology (*continued follow up)	13 years	3 years	4 years	4 years	*9 months

TINU syndrome is likely under-diagnosed meaning that prevalence figures are likely underestimated. Interestingly, in this case series, 80% of the cases were male and 20% were female. This male predominance lies in contrast to published research [2]. The average age of TINU diagnosis in this case series was 54.8 years. Also, this lies in contrast to reported literature where Mandeville et al. reported a median age of 15 years in their cohorts [2]. A potential explanation for this is the low coverage surrounding this likely under-reported condition. In this study, the patient cohort was taken from a nephrology database which accounts for the slightly older mean age of patients compared to that reported in the literature. There were no paediatric cases in this particular series. The five patients in our case series met the diagnostic gold standard of documented renal biopsy reports.

One of the major challenges in TINU cases lies in the nature of elucidating the underlying cause. The causes of TIN fall into one of the following categories: iatrogenic, genetic susceptibility, infection-associated and auto-immune. TINU syndrome is typically characterised by TIN with bilateral sudden onset anterior uveitis accompanied with the characteristic features of eye pain, conjunctival redness and photophobia.

In most cases, TINU appears to be an idiopathic immune-mediated process, but it may be precipitated by drugs or infections [6]. There is currently limited scientific evidence of a causal relationship between drugs or infections and TINU but the majority of studies in the area are retrospective. A lot of the risk factors possibly identified with TINU are also very common in the general population and therefore may just co-exist with the disease [7]. In this case series, drugs and infections were excluded as causes for TINU syndrome.

A review was conducted by Mandeville et al. in 2001. Risk factors for acute interstitial nephritis were addressed for 122 of the 133 reported cases of TINU syndrome that were reviewed. Approximately one-half (59 of 122) of the cases had no reported risk factors for acute interstitial nephritis. Antecedent drug use was the most identified risk factor. Antibiotic use was documented for 29 of 122 patients (24%). Prior use of non-steroidal anti-inflammatory drugs was reported for 22 patients (18%) [2]. One case series based in Finland conducted by Perasaari et al. showed that 19/31 patients had taken antibiotics or NSAIDs within the two months leading to diagnosis [8]. In this case series, the medication lists were scrutinized for such offending agents with none showing contributory findings.

In TINU syndrome the most commonly concomitant infections originate in the respiratory tract. Other infections that have been identified in patients presenting with TINU syndrome include Epstein-Barr virus, Chlamydia trachomatis, Mycoplasma tuberculosis, Toxoplasma gondii and Varicella zoster virus reactivation [6]. Our five cases produced negative results for infectious aetiology.

Genetic markers have proven a strong association between HLA subtypes and TINU syndrome. Goda et al. discovered associations with HLA- A2, HLA- A24, HLA- A31 and HLA- DR4 subtypes among a Japanese cohort [9]. Levinson et al. found that HLA-DQA101, HLA-DQB105, and HLA-DQB101 were associated with TINU and concluded that HLA-DQA101/HDQB105 may be related to the risk of development of the disease in their population [10].

Uveitis is a clinical condition marked by intraocular inflammation which affects the uvea or middle layer of the eye. This comprises of the iris, ciliary body and choroid respectively. It accounts for 10–20% of blindness in the USA and Europe with estimates of 25% in the developing world [11]. The specific aetiology is complex with inflammatory, infective and idiopathic associations considered. The precise cause remains undetermined in up to 45% of cases [12].

Anterior uveitis represents the most prevalent form of the disease state with approximately 50% of cases reported with posterior the least common [13]. It is more common than the posterior segment form and is considered less serious. The classification system for uveitis is based on the SUN criteria which relates to standardised uveitis nomenclature. The anterior form presents as unilateral red eye with pain or photophobia with anterior chamber activity and/or FLARE. Photophobia and pain are related to ciliary muscle spasm with a dull, aching pain reported. Another feature of uveitis is keratic precipitates (KP’s) which are divided into granulomatous and non-granulomatous subtypes. These are cellular deposits which lie on the corneal endothelium. Fine KP’s are suggestive of non-granulomatous inflammation while large mutton fat deposits are consistent with granulomatous inflammation. All of the patients in this case series were diagnosed with the anterior form of uveitis which is in line with published literature.

Corticosteroids represent the cornerstone therapy for ocular inflammation and first line treatment in anterior uveitis. The topical route is preferred in uveitis cases with intravitreal reserved for more severe cases. Corneal contact time and dosing frequency play a major role in efficacy here. Close monitoring is imperative with topical steroids as there is a risk of elevated IOP, corneal epithelial toxicity and greater susceptibility to infections. Intravitreal steroids such as triamcinolone are used in the management of intermediate and posterior uveitis. Their effects last up to 8 weeks and often necessitate long-term follow-up [14]. Treatment for TIN will typically require immunosuppressive agents unless remission occurs spontaneously. Immunomodulatory therapy generally consists of oral corticosteroids such as prednisone or prednisolone in the 1- to 1.5 mg/kg dosage range. The duration and schedule for tapering of steroid dosage rest on how well and how rapidly the patient responds to treatment [15].

Immunosuppressant therapy are considered in events where corticosteroid agents prove ineffective and treat frequent flares and chronicity. A review of the current literature reveals that methotrexate, mycophenolate mofetil and azathioprine are regarded as safe and effective for managing TINU syndrome [16]. The findings report that TNF-alpha inhibitors represent a viable treatment alternative for patients refractory to systemic corticosteroids and DMARD (Disease modifying anti-rheumatoid drug) therapy alike [17]. TNF-alpha inhibitors which include adalimumab and infliximab are used in cases unresponsive to monotherapy with agents which include DMARDs, methotrexate and mycophenolate mofetil [18]. The evidence in the literature is significant for case study and case series level only with a dearth of randomized controlled trial level evidence meaning that there are no formal clinical protocols available. In this regard, there is a noteworthy role for further research into the development of clinical protocols aimed at the patient cohort who prove refractive to corticosteroid therapy.

One of the limitations of this study is that it is retrospective in nature. However, this study is the first of its kind in the republic of Ireland that captures patients with a proven renal biopsy result significant for tubulointerstitial nephritis (TIN) in a defined population cohort. These patients all received long-term follow-up through the nephrology and ophthalmology departments which provided reports on long-term visual outcomes and treatment over extended time periods. Bilateral uveitis typically warrants a series of investigations including a vasculitic screen and rheumatology panel. However, the nephrology causes are often neglected and this article aims to highlight the importance of testing for rare causes of bilateral anterior uveitis.

In conclusion, TINU syndrome is a rare and likely under-diagnosed condition. The key learning point is the necessity to add in extra investigations in the clinical context of bilateral uveitis. Clinical relationships typically exist between ophthalmology and other medical specialties such as rheumatology and neurology. However, this article cleverly articulates an evolving association between ophthalmology and nephrology with extended scope for further collaboration with regard to likely missed cases. Specifically, a serum creatinine and urea level in addition to β2 microglobulin are relevant markers as to consider a renal component to the disease state. This would prompt a close relationship between ophthalmology and other clinical specialties. In summary, TINU is a rare and likely under represented clinical condition and this article highlights the importance of an open minded approach to assessing bilateral anterior uveitis.

## Data Availability

No datasets were generated or analysed during the current study.
